# Tailoring Intrinsic Properties of Polyaniline by Functionalization with Phosphonic Groups

**DOI:** 10.3390/polym12122820

**Published:** 2020-11-27

**Authors:** Beatriz Martínez-Sánchez, Diego Cazorla-Amorós, Emilia Morallón

**Affiliations:** 1 Departamento de Química Física and Instituto Universitario de Materiales de Alicante (IUMA), University of Alicante, Ap. 99, 03080 Alicante, Spain; beatriz.ms@ua.es; 2 Departamento de Química Inorgánica and Instituto Universitario de Materiales de Alicante (IUMA), University of Alicante, Ap. 99, 03080 Alicante, Spain; cazorla@ua.es

**Keywords:** phosphonated polyaniline, self-doping, phosphonic acid, copolymerization

## Abstract

Phosphonated polyanilines were synthesized by copolymerization of aniline (ANI) with both 2- and 4-aminophenylphosphonic acids (APPA). The material composition and the final properties of the copolymers can be easily tailored by controlling the monomers ANI/APPA molar feed ratio. An important influence on the reactivity of monomers has been found with the substituent position in the ring, leading to differences in the properties and size of blocks of each monomer in the polymer. As expected, while 2APPA shows more similarities to ANI, 4APPA is much less reactive. Phosphorus loading of ~5 at% was achieved in the poly(aniline-*co*-2-aminophenylphosphonic acid) (PANI2APPA) with a 50/50 molar feed ratio. All the resulting copolymers were characterized by different techniques. Experimental results and density functional theory (DFT) computational calculations suggest that the presence of phosphonic groups in the polymeric chain gives rise to inter- and intra-chain interactions, as well as important steric effects, which induce a slight twist in the substituted PANI structure. Therefore, the physicochemical, electrical, and electrochemical properties are modified and can be suitably controlled.

## 1. Introduction

Polyaniline (PANI) stands as one of the most attractive and revolutionary conjugated conducting polymers (CPs), which has led to an intense scientific research since its discovery in the 1980s [1,2]. Particularly, its environmental and thermal stability, low-cost and ease of synthesis, high conductivity upon doping with acid, structure diversifications and unique doping/dedoping chemistry [2] give rise to a wide applicability, e.g., in energy production and storage devices [3,4,5], biosensors [6], electrochromic devices [7] or as a precursor of heteroatom-doped carbon-based materials [8,9,10].

Interestingly, most of the recent research uses PANI to obtain hybrid materials with synergetic effects, especially with carbon nanostructured materials [11,12,13] and metal nanoparticles as well [14]. Nevertheless, to fully exploit its outstanding properties, the chemical modification of PANI is commonly employed as a strategic method to break down its poor solubility in common solvents and extend its operative pH range, driving towards better processable multifunctional materials. Concerning the synthesis, three main approaches have been widely developed to prepare the modified PANI, either the use of functionalized protonic acids as dopant, the post-synthetic modification of the pristine polymer, or the use of adequate substituted derivatives of aniline as a monomer [15,16]. The chemical and the electrochemical incorporation of new functional groups into the PANI structure has received strong interest in many fields [17].

The improvement in solubility in the presence of substituent groups is mainly ascribed to further compatibility between the polymer and solvent by decreasing the rigid interchain interactions. In addition, inserting acidic groups in the hydrocarbon chain can help keep the electroactivity at higher pH values, since the local pH of the nitrogen atoms is shifted [18]. However, in many cases, the functionalized PANI yields significantly lower electrical conductivity compared with the parent PANI. This is normally attributed to the decreased conjugation distribution and impaired mobility of charge carriers due to inductive and/or steric effects. Steric effects may also result in a total hindrance to the homopolymerization of substituted monomers. To overcome these concerns, copolymerization with aniline is considered as a key alternative, in which the material composition and the final properties can be easily tailored by a suitable controlling of the monomers ratio in the feed solution [16,19,20]. In this sense, it is of great importance to consider the relative reactivity of each monomer and the influence of the substituent position in the ring, in order to more accurately predict the degree of modification of the resulting copolymer [19]. This technique successfully allows reaching a compromise between the solubility, electrical conductivity, and electroactivity of the obtained polymer.

The sulfonation of PANI with sulfonic acid groups was one of the first contributions in the research of soluble and self-doped conducting polymer derivatives, obtained by Yue and Epstein in 1990 [21]. Since this work, several studies have been published, dealing with the functionalization of PANI backbone with different modifying moieties [22,23,24,25,26], either on the aromatic ring or the secondary nitrogen atoms. Likewise, PANI bearing acidic phosphonic groups are particularly promising, since the two available acidic protons give them additional properties for specific applications, such as acid/base complexation. It is also noteworthy that phosphonic acid doped emeraldine salts reveal a higher environmental and thermal stability, with nonvolatile dopant moieties [27], as well as a high proton conductivity due to its self-dissociation nature [28]. Interestingly, phosphorus species can act as anchorage points for metallic nanoparticles or for the immobilization of enzymes, which has been of special interest in electrocatalysis, biomedicine, and biocatalysis applications [29,30,31,32]. Regarding fuel cell applications, the incorporation of phosphonic acid units in a polymeric backbone is expected to produce promising candidates as proton exchange membranes, in order to enhance the thermal and oxidative stability, as well as ionic conductivity. Furthermore, phosphonated aromatic polymers have revealed a better behavior for this purpose than with the presence of other acidic groups, such as sulfonic groups [33]. Despite such attractive features, research on ring-phosphonated polyanilines is still limited. Chan et al. [27,34] studied the use of alkyl- and benzyl- phosphonic acids as dopants in the PANI system, both by post-modification of PANI-base and by oxidative coupling of phosphonated anilines, although phosphonic acid groups were not directly attached to the aromatic rings in any case. Amaya et al. [35] evaluated the direct linkage of phosphonic moieties in the PANI backbone via oxidative polymerization. However, methoxy- moieties were required to achieve the proper reactivity of monomers, leading to additional functionalities to the phosphonic group. A phosphonic acid ring-substituted PANI free of other functionalities was successfully attained via reductive phosphonation of pernigraniline and subsequent hydrolysis [36]. Other approaches in the literature involving the phosphonation of PANI-derived structures [29,37], as well as its subsequent carbonization [38] have also been studied. In a previous study, we carried out the electrochemical copolymerization of phosphorus-containing polyanilines thin films on a platinum electrode [39]. However, to the best of our knowledge, no selective and controlled chemical copolymerization of anilines with phosphonated anilines has been studied yet.

In the present study, we focus on the chemical synthesis and characterization of copolymers of aniline with 2- and 4-aminophenylphosphonic acids (APPA) at different feed molar ratios. The degree of phosphonation can be controlled with the monomer feed molar ratio and the substituent position in the ring of APPA. The resulting functionalized materials were characterized by X-ray photoelectronic spectroscopy (XPS), Fourier-transform infrared spectroscopy (FTIR), electrochemical impedance spectroscopy (EIS), and ultraviolet-visible (UV-Vis) spectroscopy. The electrochemical properties of the copolymers were analyzed at different pH solutions by cyclic voltammetry. In addition, density functional theory (DFT) computational calculations were also performed for a better understanding of the distribution of monomers in the polymer structure and the effect of phosphorus functionalities on its physicochemical, electrical, and electrochemical properties.

## 2. Experimental

### 2.1. Reagents

The aniline (ANI) ACS reagent (≥99.5%) was obtained from Sigma-Aldrich (Merck, Darmstadt, Germany). Prior to its use, it was distilled in a vacuum in order to remove oligomeric products generated during storage at low temperature (about 4 °C). The phosphonated monomers 2- and 4-aminophenylphosphonic acid (2APPA and 4APPA, respectively; ≥95%) were supplied by Chem Space (Riga, Lithuania). Ammonium peroxydisulfate (APS, (NH_4_)_2_S_2_O_8_; p.a.) was obtained from Merck (Darmstadt, Germany). The hydrochloric acid (HCl, 37%) was purchased from ITW Reagents (PanReac AppliChem, Barcelona, Spain). Ammonium hydroxide (NH_4_OH, p.a.) was provided by Sigma-Aldrich. Potassium hydroxide (KOH; 85%) and dipotassium hydrogen phosphate trihydrate (K_2_HPO_4_·3H_2_O) were supplied from VWR Chemicals Prolabo (Lutterworth, Leicestershire, UK). Potassium dihydrogen phosphate (KH_2_PO_4_) was purchased from Merck (Darmstadt, Germany). All aqueous solutions were prepared with an ultrapure water (18.2 MΩ·cm, Millipore^®^ Milli-Q^®^ water, Merck, Darmstadt, Germany). Nitrogen (N_2_; 99.999%) and hydrogen (H_2_; 99.999%) gases were supplied by Air Liquide. Potassium bromide (KBr; p.a.) was obtained from Merck (Darmstadt, Germany) and was used to form pellets for FTIR measurements.

### 2.2. Chemical Synthesis

Polyaniline (PANI), poly(aniline-*co*-2-aminophenylphosphonic acid) (PANI2APPA), and poly(aniline-*co*-4-aminophenylphosphonic acid) (PANI4APPA) were synthesized by chemical oxidation through adding an oxidizing agent (ammonium peroxydisulfate) in an equimolar ratio with the monomers in the aqueous acid solution (HCl, 1 M). This method was based, in part, on previously described procedures for polyaniline synthesis [1]. Two different molar ratios of aniline and phosphonated aniline (80/20 and 50/50 of ANI/APPA) were added in the feed solution, with a total concentration of 0.1 M. Throughout the copolymerization, the mixture was kept under vigorous stirring at ca. 0 °C in an ice bath. After 3 h, the polymer precipitate was collected by vacuum filtration (Membrane Filters—Nylon 0.45 μm, Teknokroma, Barcelona, Spain). This process is followed by washing several times with a 1 M HCl solution (doping process) and ultrapure H_2_O, which were previously cooled to avoid the loss of polymer by solubilization. The resulting powder was dried under a dynamic vacuum for ca. 24 h at room temperature.

### 2.3. Copolymer Characterization

#### 2.3.1. Spectroscopic Analysis

FTIR spectra were measured in a Nicolet 5700 spectrometer (Thermo Electron Corporation, Waltham, MA, USA) equipped with a Mercury-Cadmium Telluride (MCT) detector cooled with liquid nitrogen and at 4 cm^−1^ resolution. All the polymers in their salt form were prepared in KBr pellets by applying a constant force of 5 tons for 5 min. The FTIR spectra were expressed as absorbance.

X-ray photoelectron spectroscopy (XPS) experiments were performed in a VG-Microtech Multilab 3000 electron spectrometer (VG Scientific, Sussex, UK) equipped with a semispherical electron analyzer with nine channeltrons (passing energy of 2–200 eV) and an X-ray source with Al radiation (Kα 1253.6 eV). The bond energy of the C1s peak at 286.4 eV was taken as an internal standard. The deconvolutions of the P2p and N1s peaks were done by minimum squares fitting using Gaussian-Lorentzian curves, while the Shirley method was used for background determination. The full-width at half-maximum (FWHM) was maintained between 1.4 and 1.7 V. The P2p spectra were analyzed considering the spin-orbit splitting into two doublet peaks (P2p^3/2^ and P2p^1/2^) with a 2:1 peak area ratio and separated by 0.87 eV.

#### 2.3.2. Copolymer Composition

The behavior of monomers in the copolymerization is especially useful to study the effect of the chemical structure on the reactivity and copolymer composition. The ratio of aniline (ANI) and aminophenylphosphonic acid (APPA) in the copolymer was estimated using the P/N atomic ratio obtained by XPS, taking into account that P comes only from APPA monomers, whereas N is present in all of them (ANI and APPA). Therefore, it is assumed from their theoretical composition that ANI homopolymers have a P/N atomic ratio of zero (P/C = 0), whereas in APPA homopolymers the P/N atomic ratio is equal to 1 (P/C = 0.167). The monomers reactivities were also calculated by fitting the experimental data using the Solver analysis tool in the Excel^®^ program (Microsoft 365^®^) from the following equation:

(1) $$F_{\mathrm{ANI}}=\frac{r_{\mathrm{ANI}}{\left(f_{\mathrm{ANI}}\right)}^{2}+f_{\mathrm{ANI}}\;f_{\mathrm{APPA}}}{r_{\mathrm{ANI}}{\left(f_{\mathrm{ANI}}\right)}^{2}+2{\;f}_{\mathrm{ANI}}\;f_{\mathrm{APPA}}+r_{\mathrm{APPA}}\;{\left(f_{\mathrm{APPA}}\right)}^{2}}$$

**Scheme 1:**
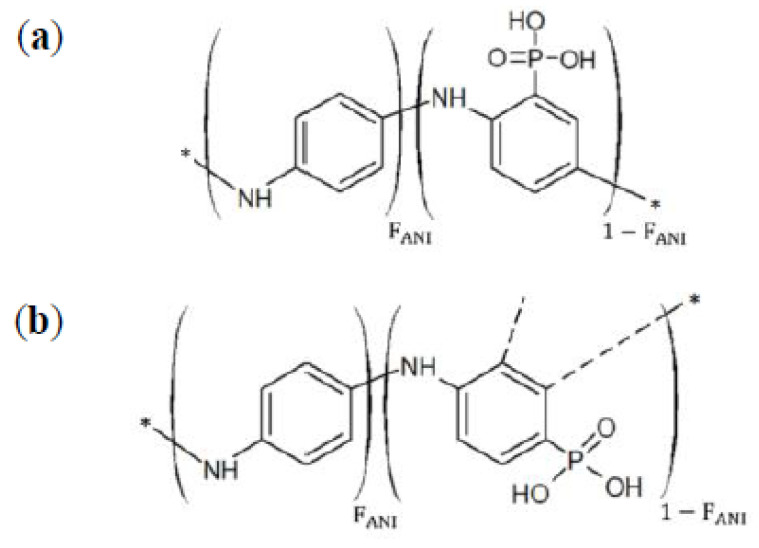
Chemical structures for: (**a**) PANI2APPA and (**b**) PANI4APPA copolymers.

This equation is deduced from the first order Markov or terminal model of copolymerization [40], where F_ANI_ is defined as the molar fraction of aniline units in the copolymer and f_ANI_ as the molar fraction of aniline in the feed. Likewise, the molar fraction of aminophenylphosphonic acid in the feed (f_APPA)_) can be expressed as 1—f_ANI_, r_ANI_, and r_APPA_ and correspond to aniline and aminophenylphosphonic acid reactivity parameters, respectively. A general structure for the PANI2APPA and PANI4APPA copolymers is shown in Scheme 1.

From the reactivity calculation (Equation (1)), it is possible to estimate the size blocks of each co-monomer (Equation (2)):

(2) $$N_{\mathrm{ANI}}=r_{\mathrm{ANI}}\frac{f_{\mathrm{ANI}}}{f_{\mathrm{APPA}}}$$

where N_ANI_ is the average number of aniline units in a polymeric block. The same equation with the corresponding data is used to obtain the estimated value of aminophenylphosphonic acid units in a polymeric block (N_APPA_).

Here, it should be noted that the first order Markov or terminal model is only valid for low conversions and the calculated reactivities could differ from the actual values. This effect is caused by differences in the consumption rate of the two monomers along the polymerization time. The most reactive monomer preferentially participates in the formation of the polymerization nuclei and, consequently, the effective monomer feed ratios and the copolymer composition will change with the conversion (compositional shift) [40]. However, within the scope of this study, the main outcomings can be drawn without the need for precise reactivity values. Furthermore, a previous study of copolymers with aniline and substituted anilines compared the terminal model with the penultimate explicit model and confirmed that the first one seems to fit better the experimental data for f_ANI_ higher to 0.3 [26], then valid for our study. The homogeneity of the material, both surface and bulk, without further hydrolysis of phosphorus moieties belonging to APPA, is also assumed.

#### 2.3.3. Solubility Testing

The solubility of copolymers in H_2_O was evaluated. A known amount of each sample in powdered doped form was added to 3 mL of solvent and stirred for 1 h. Afterwards, the non-soluble fraction was collected by filtration (Membrane Filters—Nylon 0.45 μm, Teknokroma, Barcelona, Spain) and dried under a dynamic vacuum for ca. 24 h. The filter paper was weighed before and after the filtration and drying step to calculate the solubility of copolymers. The amount of dissolved copolymer has been calculated by difference.

#### 2.3.4. Electrochemical Characterization

The electrochemical characterization of the functionalized PANIs was performed by cyclic voltammetry using an Autolab PGSTAT 302 (Metrohm, Netherlands) potentiostat. The electrochemical setup was a standard three-electrode cell, where a reversible hydrogen electrode (RHE) immersed in the working solution was employed as the reference electrode (RE). A platinum wire was used as a counter electrode (CE). The working electrode (WE) was prepared using glassy carbon (GC, 3 mm of diameter) as support, modified by dropping 20 μL of 1 mg mL^−1^ dispersion of each copolymer in a 1 M NH_4_OH aqueous solution. Three deoxygenated aqueous solutions with different pH values were employed as electrolytes: Acid medium (1 M HCl), neutral medium (0.1 M PBS, pH = 7.2), and alkaline medium (0.1 M KOH). All the characterizations were performed in a potential range from 0 to 1.2 V at 50 mV s^−1^.

#### 2.3.5. Electrochemical Impedance Spectroscopy

The Electrochemical Impedance Spectroscopy (EIS) analysis was performed at 0.6 V vs. RHE. Data were collected in the frequency range of 10 mHz to 100 kHz with an amplitude for the voltage signal of 10 mV in a 1 M HCl solution. EIS experiments were carried out in a three-electrode cell. All the potentials have been referred to the reversible hydrogen electrode (RHE).

#### 2.3.6. UV-Vis Analysis

UV-Vis spectra of PANI and all copolymers were measured in 1 M NH_4_OH aqueous solutions on a V-670 UV-Vis-NIR spectrophotometer by Jasco (Tokyo, Japan), using a wavelength range of 220–1100 nm.

#### 2.3.7. Thermogravimetric Analysis

Thermogravimetric analyses (TGA) were performed on a DSC-TGA equipment (simultaneous TGA/DSC SDT Q600, TA Instruments, New Castle, DE, USA). Around 5 mg of the dried sample was put in the thermobalance. The thermobalance was purged for 45 min under the He flow rate of 100 mL min^−1^ and then heated up to 800 °C (heating rate 10 °C min^−1^).

#### 2.3.8. Computational Calculations

Computational chemistry [41] makes use of mathematical models to simulate interactions between atoms and thus, to explain experimentally observed results, which provide us new insights and knowledge with predictive values. In this work, Avogadro (version 2.0.8.0) was used to obtain the proposed structures and for the optimization of the geometry. The electronic density and the energy of the systems were determined using the Gaussian 09 program through the density functional theory (DFT) methodology with a functional B3LYP and 6-31G(d) basis set [42], where self-consistent-field (SCF) energies correspond with free energies. Furthermore, the IR spectra of the proposed structures have been obtained by vibrational frequency calculations, testing that the final geometries are true stationary points.

## 3. Results and Discussion

### 3.1. Copolymer Composition

The copolymerization of aniline with aminophenylphosphonic acids was successfully carried out by the chemical oxidation procedure. Two different molar ratios were added in the feed solution, 80/20 (f_ANI_ = 0.8) and 50/50 (f_ANI_ = 0.5). An attempt of copolymerization with a higher molar fraction of 2APPA (20/80 of ANI/2APPA) was carried out without success, probably due to steric effects that hinder the proper polymerization, thereby obtaining soluble oligomers. Therefore, the homopolymerization of APPA monomers was not performed.

The composition of the synthesized copolymers has been estimated by the XPS analysis, following the equations described in the Experimental section. Table 1 shows the atomic percentages of PANI2APPA and PANI4APPA, together with those of PANI synthesized under the same conditions for comparison purposes. The P/N atomic ratio sets the upper limit to the level of P-incorporation and it is used to estimate the fraction of phosphonated monomer incorporated into the copolymer. Interestingly, important differences in the modification level can be clearly observed with the position of the amino group in the aminophenylphosphonic acid. In this sense, the higher the 2APPA molar fraction in the feed, the higher the phosphorus content detected in the samples, affording a P-loading of ~5 at% (~12 wt%) for PANI2APPA (50/50). Nonetheless, if 4APPA is used, the degree of phosphorus incorporation becomes less than half for the same APPA feed molar ratio (50/50). Since each phosphonic group (R-PO(OH)_2_) has three oxygen atoms, the O/P atomic ratio obtained by XPS confirms that this proportion is approximately maintained in the copolymers. The O content of the PANI has been subtracted for this calculation. This oxygen content can be explained by over-oxidation of the polymer chain. However, a small contribution of phosphate groups (R-O-P(OH)_2_) and/or O incorporation by over-oxidation of the polymer cannot be ruled out.

The relationship between the composition in the feed (f) and the resulting copolymers (F) is shown in Table 2. The table also includes the reactivity ratios and the average number of units of each monomer in a polymeric block. The results show that, in PANI2APPA copolymers, both the reactivity of ANI and 2APPA are close to unity. When two monomers have the same preference for adding into the copolymer (r_ANI_ = r_APPA_ = 1), the reaction is known as an ideal copolymerization and the copolymer composition is the same as the co-monomer feed (f_ANI_ and F_ANI_) [40], as observed in Table 2. On the contrary, the two monomer reactivities for PANI4APPA and ANI are significantly different, due to the fact that the reactivity of ANI is much higher than 4APPA. This effect is probably due to the impossibility of 4APPA to couple with other free monomers or oligomers through the head-to-tail mechanism, since the *para-* position is blocked, giving rise to important steric effects. Accordingly, the resulting polymer is more likely to have long linear sequences of PANI combined with localized crosslinks or irregularities due to 4APPA. Therefore, it has been confirmed that the position of the amino group in the aminophenylphosphonic acid is a determining factor in the reaction pathway. Consequently, the arrangement of the ANI/APPA units into the polymer chain and the block size of aniline (N_ANI_) are also affected. When comparing these results with those obtained for sulfonated polyaniline under the same synthesis conditions, significant differences are observed related to the reactivity of monomers, in which sulfonated monomers reveal a much lower reactivity than APPA [25]. Therefore, the degree of incorporation of APPA into the polymeric chain is higher, especially when 2APPA is used.

**Figure 1:**
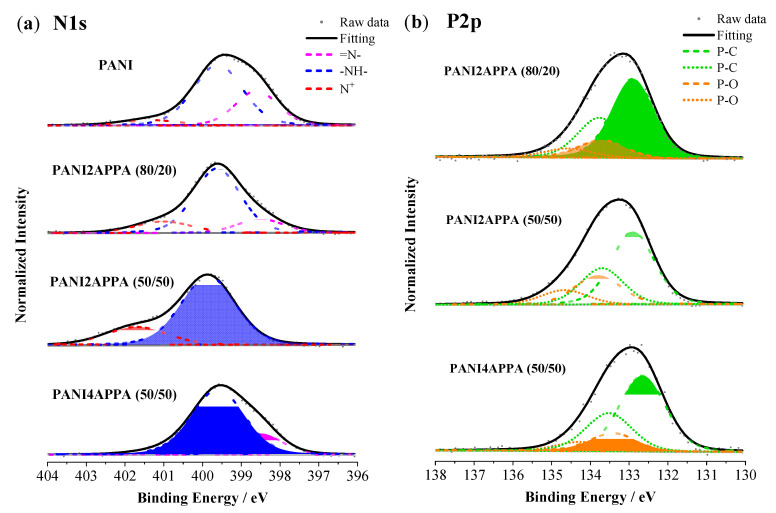
(**a**) N1s and (**b**) P2p spectra of PANI, PANI2APPA feed ratio 80/20 and 50/50, and PANI4APPA feed ratio 50/50.

**Scheme 2:**
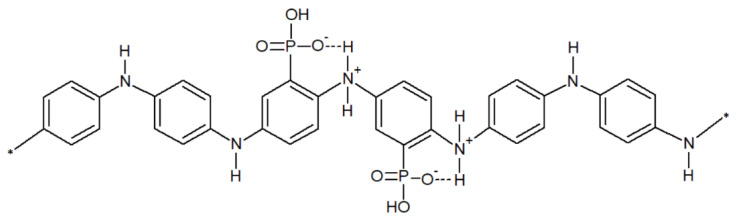
Structure formed in PANI2APPA (50/50) by electrostatic effects.

In order to get a better understanding of the nature of the phosphonated polyanilines, deconvoluted N1s and P2p spectra are shown in Figure 1. The N1s core-level spectra were fitted with three main contributions, which could be assigned to distinct nitrogen species, showing differences depending on the feed ratio and APPA isomers employed. The first and second peak are observed at lower binding energies, at around 398.5 and 399.5 eV, and can be attributed to the presence of neutral imine and amine-type nitrogen, respectively. These N-species are commonly found in PANI chains [43]. Furthermore, a third contribution at higher binding energies (401.1–401.7 eV) can be associated with positively charged N sites (N^+^), such as radical cation (–N^•+^–) or protonated nitrogen (–NH_2_^+^–, –N^+^H=) [36]. Table 1 shows the ratio between charged and neutral nitrogen species (N^+^/N), which can be related to either the doping efficiency of the copolymers or the charge stabilization. Interestingly, the higher the concentration of 2APPA units (F_2APPA_), the higher the N^+^/N ratio, in which no imine species (=N–) are found, indicating their full protonation. These results suggest that the strongly acidic character of the phosphonated functionalities results in a higher positive charge localization on the nitrogen atoms, either polaronic or bipolaronic. Such localization effects have been already observed in other studies [19,21,25] by a strong inter- and intra-chain coulombic attraction between both charged species (Scheme 2) or by hydrogen bonds (N–H···O and O–H···N), which is favored when both functionalities (phosphonic and amine groups) are in an ortho position. Despite the favorable doping levels in PANI2APPA copolymers, the increased charge on the nitrogen sites could lead to an important drop in conductivity with respect to conventional acid-doped polyanilines. Other important factors can also affect the conductivity, e.g., the steric reduction of chain coherence and the increase in chain free-volume due to large functional groups covalently attached, as well as the higher concentration of defects along the chain [34]. Consistent with this discussion, the low incorporation of phosphonated moieties in PANI4APPA causes the doping level and probably the conductivity to remain unchanged from the pristine PANI. Nevertheless, when sulfonated polyanilines are analyzed under a similar molar composition in the resulting copolymer [25], a higher self-doping degree is shown than with APPA, thus revealing that sulfonated groups seem to have a greater doping capacity.

For all the copolymer samples, a visible dissymmetry in the P2p spectra shapes suggests the presence of two different P contributions formed by two asymmetric doublets. The first one at 132.7 eV lies in the range of binding energies for P–C compounds, which can be related to the phosphonic group incorporated to the polyaniline structure. A second contribution, much less intense, appears at around 133.7 eV, which is characteristic of P–O species [44,45] and might be ascribed to phosphate groups (R–O–P(OH)_2_) generated during the synthesis at oxidative conditions. It is noteworthy that the P2p profiles for PANI2APPA are slightly shifted to higher binding energies with respect to PANI4APPA, suggesting some differences in the environment surrounding the phosphorus atoms as a consequence of the interaction with the nitrogen groups. In this sense, it could be suggested that, in the case of PANI4APPA, electrostatic interactions within the chain are not favored, but without ruling out the interactions between the pendent phosphonated group and nitrogen atoms of different polymeric chains. A slight increase in phosphate or phosphoric groups can be observed when a higher P-loading is reached (from 19% in PANI2APPA 80/20 to 30% in PANI2APPA 50/50). This effect can be explained by oxidation of part of the phosphonic groups to phosphoric groups, as it has been also observed in previous studies for phosphonic acid ring-substituted polyanilines synthesized via an electrochemical route when high oxidation potentials are applied [39,46]. No residual phosphorus was detected in the PANI.

### 3.2. FTIR Spectroscopy

**Figure 2:**
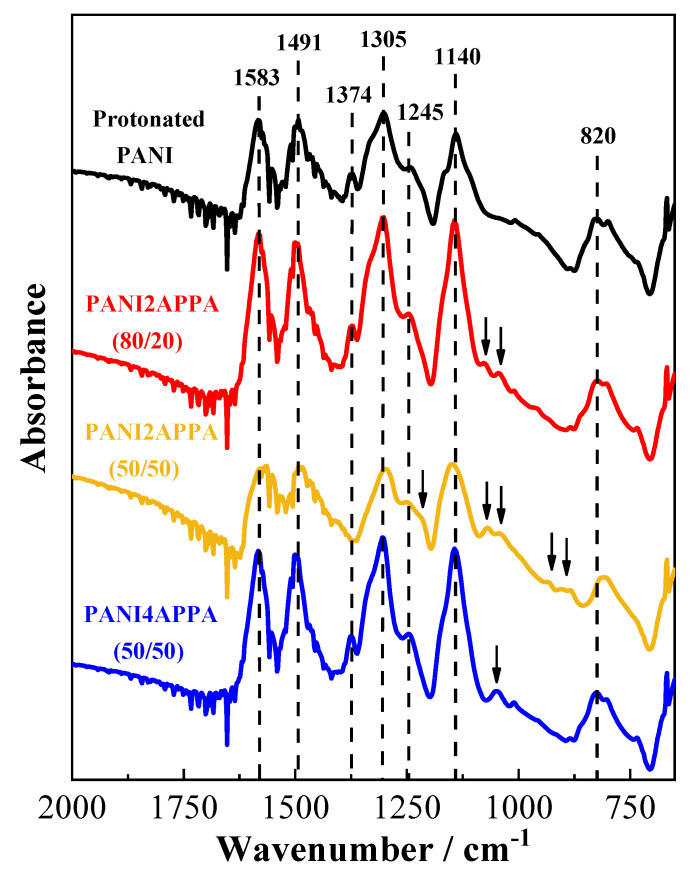
FTIR spectra obtained for protonated PANI (black line), PANI2APPA feed ratio 80/20 (red line) and 50/50 (orange line), and PANI4APPA feed ratio 50/50 (blue line).

The FTIR spectra of PANI, PANI2APPA (80/20 and 50/50), and PANI4APPA (50/50) are compared in Figure 2. In all spectra, similar vibrational modes to those described for conducting the PANI skeleton can be observed [47], with the 1583 to 1491 cm^−1^ bands associated to quinoid and benzenoid and with a band intensity ratio typical of a predominant emeraldine oxidation state. The proposed assignments for the most relevant absorption bands are listed in Table 3. Compared with PANI, additional bands appear in phosphonated copolymers (marked with black arrows). The band at 1075 cm^−1^ could be due to different contributions, including P–O–C out-of-plane stretching and P–Ar stretching [48,49]. Another band at 1040 cm^−1^ and two small contributions at 900–930 cm^−1^ are probably related to P-O stretching in O=P–OH with a single neighboring –OH group [34,48,49]. The last ones are mainly present in PANI2APPA 50/50 copolymers. Moreover, a shoulder that arises at around 1214 cm^−1^ is observed in PANI2APPA (50/50), which could be ascribed to P=O stretching [27,48]. This band is not observed in the other copolymer spectra due to the lower P content and its overlapping with the stronger amines C–N stretching.

### 3.3. Solubility

The solubility of phosphonated copolymers was assessed in H_2_O. Phosphonated groups have been successfully incorporated in the PANI chain in order to enhance its insufficient solubility in solvents commonly used, such as H_2_O, at room temperature. In this way, solubility values close to 1 g L^−1^ have been achieved for the highest P-loading (PANI2APPA 50/50, F_APPA_ = 0.53). Therefore, it suggests that the higher the P content, the higher the solubility. Interestingly, despite the lower incorporation of phosphonated groups to the polymeric structure when 4APPA is used (PANI4APPA 50/50, F_APPA_ = 0.16), its solubility (~0.1 g L^−1^) is still higher than that of PANI, which is practically negligible.

### 3.4. Electrochemical Results

Thin films of PANI and PANIAPPA copolymers were deposited onto a glassy carbon (GC) electrode (0.3 mg/cm^2^) in order to evaluate the electroactivity and the electrochemical characteristics at different pH aqueous solutions: Acid medium (1 M HCl), neutral medium (0.1 M PBS, pH = 7.2), and alkaline medium (0.1 M KOH), from 0.0 to 1.2 V (vs. RHE).

**Figure 3:**
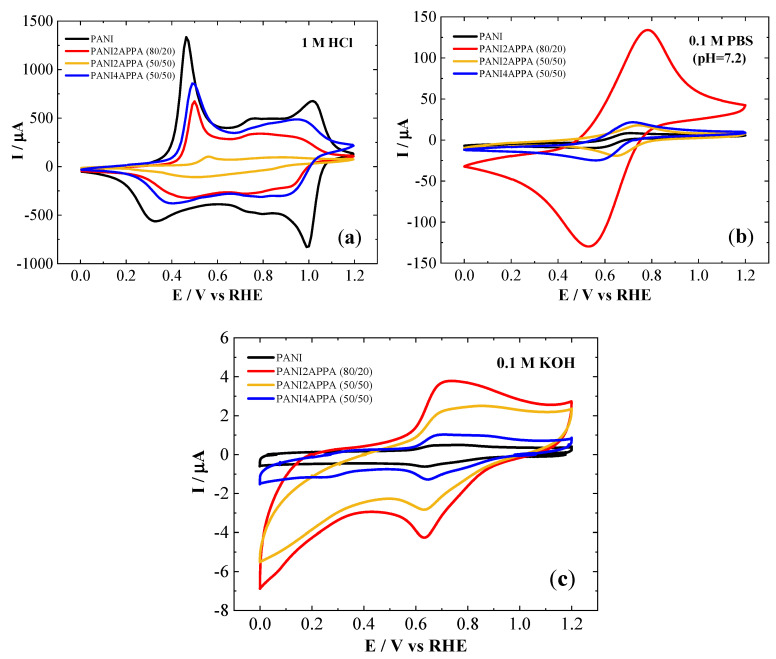
Cyclic voltammograms of Glassy Carbon (GC) electrodes modified with PANI (black line), PANI2APPA feed ratio 80/20 (red line) and 50/50 (orange line), and PANI4APPA feed ratio 50/50 (blue line) in: (**a**) Acid (1 M HCl), (**b**) neutral (0.1 M PBS, pH = 7.2), and (**c**) alkaline (0.1 M KOH) aqueous solution at 50 mV s^−1^ under N_2_ atmosphere.

The cyclic voltammograms displayed in Figure 3 reveal some differences between PANI and the phosphonated ring-substituted PANIs. In the acidic solution (1 M HCl), PANI (black lines) shows mainly two sets of redox peaks at around 0.5 and 0.9 V (vs. RHE) related to the transition between oxidation states, from leucoemeraldine to emeraldine and from emeraldine to pernigraniline, respectively [51]. In addition, the development of a third wide “middle peak” is attributed to polymer degradation reactions or crosslinked units, and it depends on multiple factors, including the monomer content, the synthetic process, or the nature of the acid electrolyte employed.

The first redox process clearly appears in all copolymers, although the peak potential is shifted to more positive potentials than in the PANI as the amount of P-loading increases, at around 30, 40, and 100 mV for PANI4APPA (50/50), PANI2APPA (80/20), and PANI2APPA (50/50), respectively. It is also highlighted that the fully reduced PANI (leucoemeraldine state) starts to oxidize at around 0.35 V towards its emeraldine state, whereas in all copolymers this oxidation is delayed by approximately the same values as the peak potential. As previously mentioned, the presence of phosphorus functionalities possibly produces inter- and intra-chain electrostatic interactions or hydrogen bonds with nitrogen groups, which withdraw electrons from the aromatic rings, making more difficult the oxidation of the N units [52]. Furthermore, steric hindrances of voluminous chain-anchored substituents must also be considered. The presence of functional groups can break the coplanarity of the oxidized units or decrease the extended conjugation by an inductive effect, affecting the conductivity and energetically unfavoring the formation of oxidized species [21].

Contrarily, the second peak is not well defined and appears less intense than in the case of PANI, or even overlapped with other middle processes. A similar behavior has been reported in other substituted polyanilines with anionic groups in the polymer backbone [25,26].

It should be highlighted that, in an acidic medium, the voltametric charge decreases as the APPA fraction increases (Figure 3a). These results support the hypothesis that the phosphorus substituents induce a twist of the PANI chain [19,36], in addition to the steric hindrance, thus shortening the effective conjugation length and affecting the electroactivity. A compromise must be reached between the chain length and the degree of phosphonation of the substituted-PANI and the electrochemical properties, making it possible to tailor the final properties of the copolymers for a given application. In addition, not only is the ANI/APPA ratio important in the final properties, but also in the distribution of APPA in the polymer backbone.

The effect of increasing the pH of the electrolyte has been also analyzed. A similar behavior is observed to that obtained with PANI in Figure 3b,c. A broad redox peak appears as a consequence of overlapping peaks, leading from the fully reduced state (leuroemeraldine) to the totally oxidized state (pernigraniline), in which the intermediate conducting emeraldine probably corresponds to a transition state in a narrow potential range [25]. However, contrary to what happens in an acidic medium, all copolymers show a higher voltametric charge than PANI, especially in the case of PANI2APPA 80/20 (F_ANI_ = 0.79). Therefore, the self-doping effect of phosphonated groups is verified by the observed electroactivity at higher pH values.

### 3.5. Electrochemical Impedance Spectroscopy

**Figure 4:**
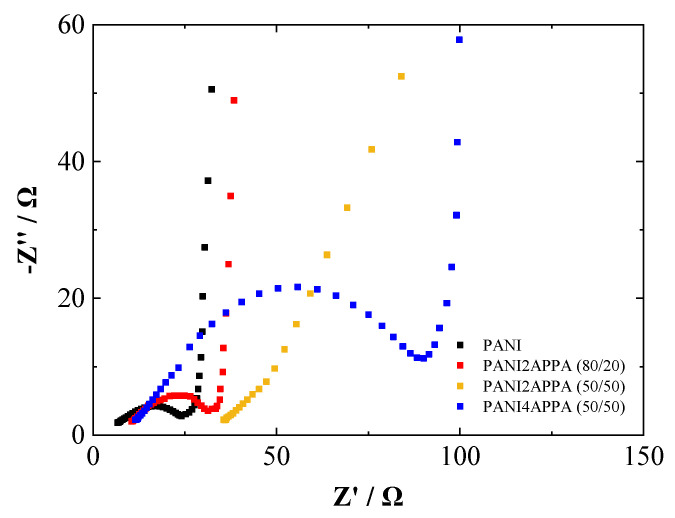
Nyquist plots of PANI (black dots), PANI2APPA feed ratio 80/20 (red dots) and 50/50 (orange dots), and PANI4APPA feed ratio 50/50 (blue dots) in 1 M HCl.

Nyquist plots for the four materials measured at 0.6 V (vs. RHE) in HCl 1 M with a polymer loading of 0.2 mg/cm^2^, are presented in Figure 4. The plots present interesting differences among the materials depending on the polymer composition. PANI has the lowest value of internal resistance (R_s_, corresponding to the first intercept of the Z’ axis). PANI2APPA (50/50) has the highest value, whereas the resistances for the other two materials are slightly higher than that for PANI (Table 4). The internal resistance is related to the resistance of the collector, the contact resistance, and the resistance of the materials. Since the collector used is the same and the electrodes consist of thin films of the materials with similar weight, the differences observed must be due to the resistance of the materials. Therefore, the results show that all synthesized copolymers have a lower conductivity than PANI and that the higher the APPA content, the lower the electrical conductivity. According to other studies, an increase in solubility usually leads to a decrease in conductivity [19,26].

The medium to low frequency region presents a semicircle, corresponding to a charge transfer process, for all the samples except for the material with the lowest conductivity (PANI2APPA 50/50). This is the sample with the lowest electroactivity as observed by CV (Figure 3a). For the two copolymers presenting the semicircle, the highest charge transfer resistance is observed for PANI4APPA 50/50 whereas PANI2APPA 80/20 has a behavior very similar to PANI. The differences in the diameter of the semicircle are an indication of differences in electrical conductivity that may affect the charge propagation. The lower conductivity of PANI4APPA 50/50 (see Table 4) may explain the higher charge transfer resistance observed for this copolymer.

The lowest frequency region for all the samples except for PANI2APPA 50/50 consists essentially of a vertical line indicating a pure capacitive behavior. However, in the case of PANI2APPA 50/50 there is no vertical line and a Warburg region is observed from a Z’ value of 50 Ω, indicating an ion diffusion resistance which must be a consequence of the high resistance of this material.

### 3.6. UV-Vis Spectroscopy

**Figure 5:**
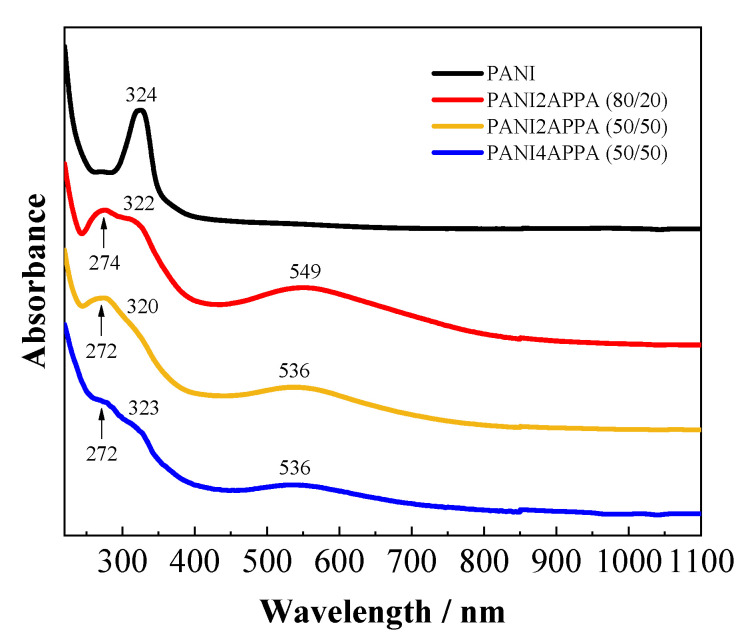
UV-Vis spectra of PANI (black line), PANI2APPA feed ratio 80/20 (red line) and 50/50 (orange line), and PANI4APPA feed ratio 50/50 (blue line) in a 1 M NH_4_OH solution.

Figure 5 shows UV-Vis spectra of PANI and synthesized copolymers in a 1 M NH_4_OH solution. Differences in the color of solutions are clearly perceptible to the naked eye in Figure S1. The PANI UV-Vis spectrum has a main band at 324 nm, which has been attributed to the $\pi_{B}\to \pi^{*}$ bandgap transition with other contributions [21,34]. The hypsochromic shift of this electronic transition from PANI to PANI2APPA copolymers is in agreement with a decrease in the extended conjugation due to a decrease in coplanarity of the π system, hindering electron hopping and increasing transition energy. Consequently, the conductivity decreases what is consistent with EIS results. This effect is also observed in sulfonated [21] and carboxylated polyanilines [19,26], and can be due to a larger steric hindrance of phosphonated groups and neighboring hydrogen in adjacent rings or strong inter- and intra-chain interactions. It should be also highlighted that this transition appears overlapped in all copolymers with a nearby absorption band at 272–274 nm, which is not observed in PANI. This band could be associated with a low-lying $\pi_{B}\to \pi_{S}$ absorption [27] or the $n\to \pi^{*}$ transition due to non-bonding electrons present in the R-PO_2_(OH)^−^ group [19,26]. Moreover, all copolymers present another band at 536–549 nm related to the $\pi_{B}\to \pi_{Q}$ exciton transition, as has been reported in other ring-phosphonated polyanilines [34]. This band is also hypsochromically shifted from the conventional PANI (at around 610 nm) [53], although it is not observed in our synthesized PANI. It has been reported that the absence of this band in PANI could be due to its oxidation state, since in the leucoemeraldine state only one chromophore is present [54]. This displacement can be explained considering a decrease in overlapping of quinoid/benzenoid units due to the increased ring torsion angle [34]. As discussed above, it seems that P functionalities in *para-* position play an important role in stronger alterations despite the lower presence of 4APPA units in the chain.

### 3.7. Thermal Stability

TGA thermograms for PANI and copolymers are shown in Figure S2. Important differences are appreciable in the TG profiles for each material. The TGA experiment performed on pure PANI presents a major weight loss at around 500 °C, which is attributed to the structural decomposition of the polymer [55]. This polymer seems to be quite homogeneous, since its thermal decomposition occurs in a narrow temperature range. However, copolymers show a different behavior, probably due to the incorporation of phosphonated functionalities that affect the polymer growth, in which shorter chains are developed together with a higher degree of heterogeneity. In fact, the TG profiles for PANI4APPA 50/50 and PANI2APPA 80/20 are quite similar to that of PANI except for the wider range of temperatures in which the decomposition occurs, in agreement with the higher degree of heterogeneity of the copolymers. Interestingly, the sample with the higher phosphorus content (PANI2APPA 50/50) shows a different profile with an additional weight loss at around 300 °C, probably associated with the decomposition related to phosphonic acid units, such as the formation of anhydrides, as reported for other phosphonated polymers [56]. In all the cases, despite starting to decompose at lower temperatures than PANI, lower degradation rates are observed for the copolymers and higher weight percentages remain at temperatures above 520 °C.

### 3.8. Computational Calculations

Computational calculations were carried out in order to better understand the distribution of ANI and APPA monomers in the structure of the synthesized copolymers. The average length segment calculated from the reactivity values of PANI2APPA and PANI4APPA (f_ANI_ = 0.5) was used to study different arrangements of monomers and to obtain the most stable geometry. A total number of 8 monomer units were used in each case for the calculations. Only phosphonic groups have been considered for simplifying the calculation interpretation.

**Figure 6:**
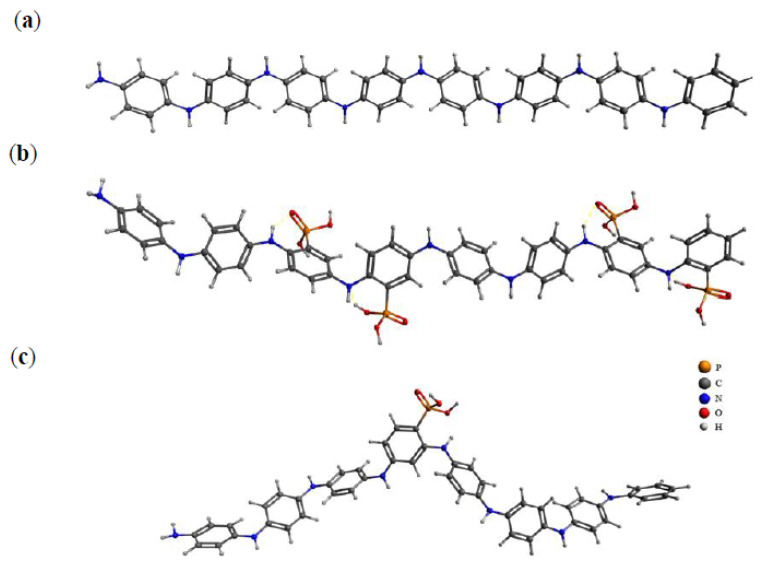
Most stable model structures for: (**a**) PANI, (**b**) PANI2APPA, and (**c**) PANI4APPA. H is white, C is grey, N is blue, O is red, and P is orange.

The energy of each polymeric system has been evaluated, optimizing their structures and checking their vibrational frequencies. Figure 6b,c shows the proposed geometries for both PANI2APPA and PANI4APPA copolymers with the lowest free energy values under the study conditions. It is observed that the incorporation of phosphonated monomers in PANI2APPA induces a slight twist of the substituted linear PANI chain, due to the *ortho-* position of the substituent holding in the polymeric backbone. In this case, intra-chain interactions between the phosphonated groups and N species likely take place (marked with yellow dashed lines), thus promoting the experimentally observed self-doping effect. In PANI4APPA, 4APPA monomers produce irregularities within a reasonably linear structure with respect to that of pristine PANI. Intra-chain interactions are probably weak or unappreciated in this case, as discussed in the XPS analysis, whereas inter-chain interactions between pendant phosphonic groups and other species are not ruled out.

The calculated IR spectra of polyaniline and proposed copolymers are displayed in Figure S3 and they can be compared with the experimental FTIR spectra (Figure 2). As can be appreciated in Table 3, small variations in the theoretical wavelength of the main bands with respect to the experimental results could be observed, since the synthesized copolymers are made up of longer chains. Furthermore, the presence of electronegative N atoms in the vicinity can also slightly shift the position of these bands. Compared with PANI, new bands are clearly appreciable in both copolymers (marked with red arrows), as a consequence of phosphonated moieties in the chain. In the two copolymers a significant band appears above 1000 cm^−1^ (at 1054 and 1074 cm^−1^ in PANI2APPA and PANI4APPA, respectively). Moreover, in PANI2APPA an intense band appears at 834–850 cm^−1^ with a shoulder at 930 cm^−1^. A shoulder at around 1221–1257 cm^−1^ also appears in copolymers, which is more evident in PANI2APPA. All these bands are due to either P=O, P–O, or P–Ar stretchings and are observed in the experimental FTIR spectra. Therefore, DFT calculations support the observed interpretation of experimental IR spectra, confirming that additional bands are related to the presence of phosphorus functionalities within the PANI chain.

## 4. Conclusions

Phosphonated ring-substituted PANIs have been successfully synthesized via the chemical oxidative copolymerization of aniline (ANI) with either 2- or 4-aminophenylphosphonic acid (APPA). Characterization of the resulting copolymers by different techniques confirms the incorporation of APPA units in the polymer chain.

An important influence on the reactivity of monomers has been found with the relative position that phosphonated groups hold in the polymer backbone, thus affecting the arrangement and the length of blocks of each monomer in the chain.

The self-doping effect of PANI2APPA copolymers was clearly observed by XPS and cyclic voltammetry, where a P-loading of ~5 at% has been achieved for a 50/50 molar feed ratio. Consequently, the solubility in H_2_O increases, but with a significant decrease in the conductivity. It should be highlighted that a moderate incorporation of P functionalities in the PANI chain (~2 at% in PANI2APPA 80/20) gives rise to a self-doped conducting polymer, highly soluble in aqueous solutions and with a good electrochemical response even at high pH values.

Despite the lower incorporation of P moieties in PANI4APPA, it seems that inter-chain interactions and steric effects play an important role in modifying its physicochemical, electrical, and electrochemical properties.

Therefore, the controlled phosphonation of PANI has been possible by the chemical polymerization method with the incorporation of a significant amount of phosphorus in the structure, providing them with promising and tunable properties and emerging as potential polymeric materials for their use in different energy and environmental applications.

## Figures and Tables

**Table 1:** Chemical composition and atomic ratios obtained from the XPS spectra of polyaniline (PANI), PANI2APPA, and PANI4APPA samples. Percentages are referred to the respective atomic total.

Sample	Atomic Formula *	N/at%	C/at%	O/at%	P/at%	P/N	O/P **	N^+^/N/%
**PANI**	C_7.4_H_x_N_1.0_O_0.3_	11.4	84.0	2.9	0.0	-	-	7
**PANI2APPA (80/20)**	C_7.1_H_x_N_1.0_P_0.2_O_0.9_	10.8	76.5	9.9	2.3	0.21	3.0	18
**PANI2APPA (50/50)**	C_6.8_H_x_N_1.0_P_0.5_O_2.0_	9.6	65.0	19.1	5.1	0.53	3.2	30
**PANI4APPA (50/50)**	C_7.6_H_x_N_1.0_P_0.2_O_0.7_	10.4	79.5	6.9	1.7	0.16	2.4	5

* Normalized to N = 1.0 (by XPS data). ** The O content of the PANI has been subtracted for the calculation.

**Table 2:** Co-monomer ratios in the feed (f), copolymers composition (F), reactivity ratios, and average length segments of PANIAPPA copolymers.

Sample	f_ANI_	F_ANI_	Reactivity		Reactivity Ratio (R_r_ = r_ANI_/r_APPA_)	Average Length Segment	
			r_ANI_	r_APPA_		N_ANI_	N_APPA_
**PANI2APPA**	0.8	0.79	1.0	1.3	0.8	5.0	1.3
	0.5	0.47				2.0	2.3
**PANI4APPA**	0.5	0.84	5.6	0.3	18.9	6.6	1.3

**Table 3:** Chemical composition and atomic ratios obtained from the XPS spectra of PANI, PANI2APPA, and PANI4APPA samples. Percentages are referred to the respective atomic total.

Wavenumber/cm^−1^		Assignments	References
Experimental	Theoretical		
1583	1550	C–C quinoid ring stretching	[47,48]
1491	1509	C–C benzenoid ring stretching	[47,48]
1374	-	C–N= in the neighborhoud of a quinoid ring	[47,50]
1305	1327–1340	C-H stretching, C–N–C stretching, or p-electron delocalization	[25,47,50]
1245	-	C–N^+^ stretching of secondary aromatic amine in the polaron structure	[25,50]
1214 *	1221–1257 *	P=O stretching	[27,48]
1140	1146	C–H aromatic bending in-plane	[47]
1075 *	1074 *	P–O–C out-of-plane stretching, P–Ar stretching	[48,49]
1040 *	1054 *	P–O stretching in O=P–OH with a single neighboring –OH group	[34,48,49]
900–930 *	930 *	P–O stretching in O=P–OH with a single neighboring –OH group	[34,48,49]
820	834–850	C–H aromatic bending out-of-plane	[50]

* Additional bands in copolymers compared with PANI.

**Table 4:** Internal resistance (R_s_) of PANI, PANI2APPA, and PANI4APPA copolymers at 0.6 V in a 1 M HCl electrolyte obtained by Electrochemical Impedance Spectroscopy (EIS).

Samples	R_s_/Ω
**PANI**	6.7
**PANI2APPA (80/20)**	10.5
**PANI2APPA (50/50)**	35.6
**PANI4APPA (50/50)**	11.6

## Supplementary Materials

The following are available online at https://www.mdpi.com/2073-4360/12/12/2820/s1, Figure S1: 10^−2^ mg mL^−1^ dispersions of: (a) PANI, (b) PANI2APPA (80/20), (c) PANI2APPA (50/50), and (d) PANI4APPA (50/50) in a 1 M NH_4_OH solution; Figure S2: TGA thermograms of PANI (black line), PANI2APPA feed ratio 80/20 (red line) and 50/50 (orange line), and PANI4APPA feed ratio 50/50 (blue line) performed under He atmosphere at a heating rate of 10 °C min^−1^; Figure S3: FTIR spectra obtained for: (a) PANI, (b) PANI2APPA, and (c) PANI4APPA using a total of 8 monomers in each case, by computational calculations.
