# Supplementary material for: Tailoring Intrinsic Properties of Polyaniline by Functionalization with Phosphonic Groups
Source: Polymers (Basel). 2020 Nov 27;12(12):2820. doi: 10.3390/polym12122820 (PMC7760660; doi:10.3390/polym12122820)
Supplement: Supplementary file 1 [file polymers-12-02820-s001.pdf]

Supplementary Materials

Tailoring intrinsic properties of polyaniline by functionalization with phosphonic groups

Beatriz Martínez-Sánchez[1], Diego Cazorla-Amorós[2], Emilia Morallón[1,*]

[1] Departamento de Química Física and Instituto Universitario de Materiales de Alicante (IUMA), University of Alicante, Ap. 99, 03080, Alicante, Spain; beatriz.ms@ua.es

[2] Departamento de Química Inorgánica and Instituto Universitario de Materiales de Alicante (IUMA), University of Alicante, Ap. 99, 03080, Alicante, Spain

* Corresponding author: morallon@ua.es (E.M.)

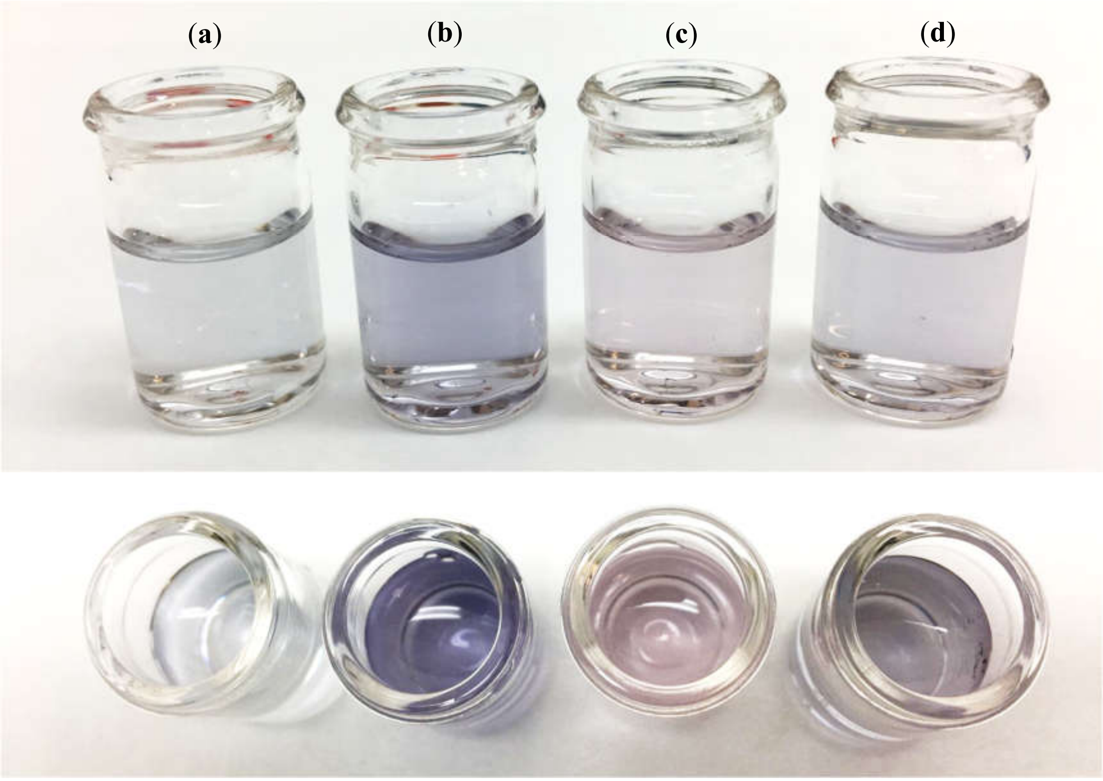

Figure S1. 10^{-2} mg mL^{-1} dispersions of: (**a**) PANI, (**b**) PANI2APPA (80/20), (**c**) PANI2APPA (50/50), and (**d**) PANI4APPA (50/50) in 1 M NH_4OH solution.

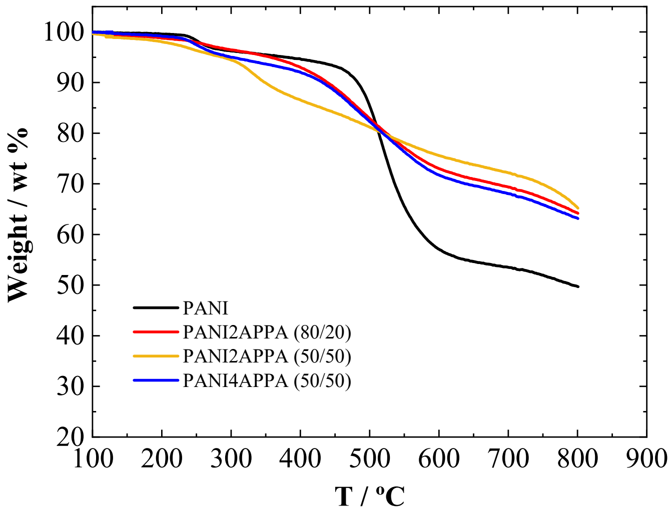

Figure S2. TGA thermograms of PANI (black line), PANI2APPA feed ratio 80/20 (red line) and 50/50 (orange line), and PANI4APPA feed ratio 50/50 (blue line) performed under He atmosphere at heating rate of 10 ℃ min^{-1}.

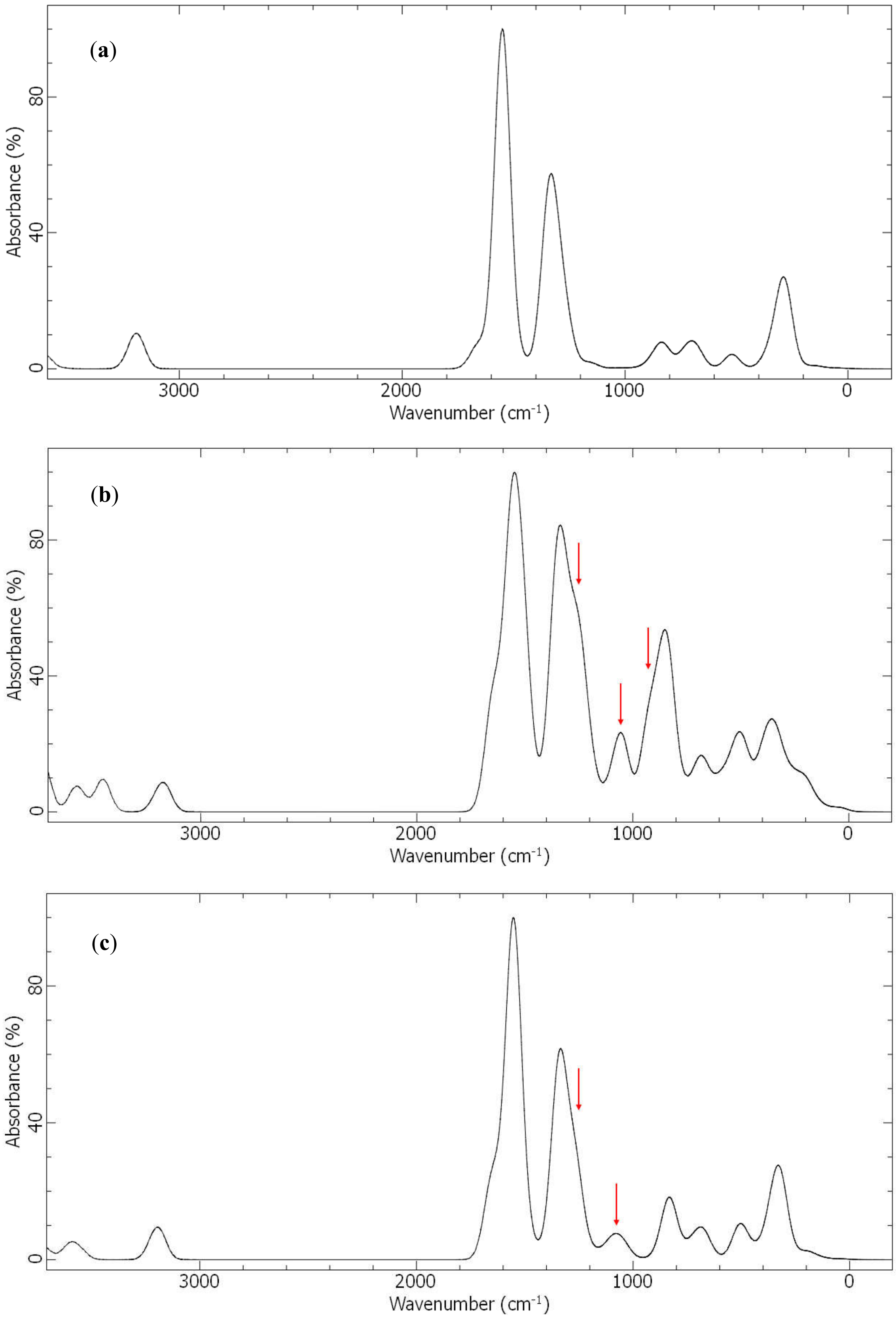

Figure S3. FTIR spectra obtained for: (**a**) PANI, (**b**) PANI2APPA and (**c**) PANI4APPA, using a total of 8 monomers in each case, by computational calculations.
